# Supplementary material for: Enhanced Clearance of Neurotoxic Misfolded Proteins by the Natural Compound Berberine and Its Derivatives
Source: Int J Mol Sci. 2020 May 13;21(10):3443. doi: 10.3390/ijms21103443 (PMC7279252; doi:10.3390/ijms21103443)
Supplement: Supplementary file 1 [file ijms-21-03443-s001.pdf]

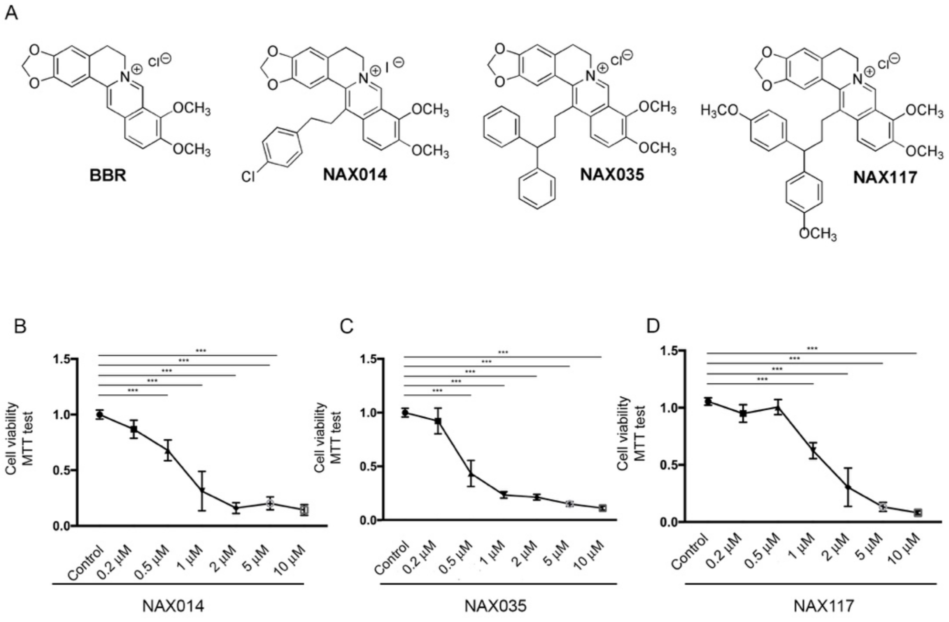

Figure S1. NAXs molecular structure and effects of NAXs on cell viability. **(A)** Molecular structures of BBR, NAX014, NAX035, NAX117. **(B-D)** MTT cell viability assay was performed on NSC34 cells treated with NAX014 **(B)**, NAX035 (**C**) and NAX117 (**D**) at different concentrations for 48 hours (*** $p < 0.001$, one-way ANOVA, followed by Tukey's test).
